# Supplementary material for: Impact of System and Diagnostic Errors on Medical Litigation Outcomes: Machine Learning-Based Prediction Models
Source: Healthcare (Basel). 2022 May 12;10(5):892. doi: 10.3390/healthcare10050892 (PMC9140545; doi:10.3390/healthcare10050892)
Supplement: Supplementary file 1 [file healthcare-10-00892-s001.zip › healthcare-1714408-supplementary.pdf]

Supplemental Table S1. Machine learning and logistic model-based prediction models for litigation outcomes in surgical patients.

	LightGBM	Decision Tree	Random Forest	Logistic Model
Accuracy	0.835 (0.796–0.875)	0.869 (0.827–0.912)	0.868 (0.824–0.911)	0.887 (0.860–0.914)
Precision	0.838 (0.793–0.883)	0.845 (0.802–0.888)	0.831 (0.786–0.875)	0.876 (0.835–0.917)
Recall	0.880 (0.840–0.920)	0.942 (0.911–0.972)	0.963 (0.934–0.992)	0.931 (0.902–0.960)
F1 score	0.857 (0.824–0.890)	0.890 (0.856–0.925)	0.892 (0.856–0.927)	0.900 (0.879–0.922)
AUC	0.899 (0.865–0.934)	0.867 (0.813–0.922)	0.896 (0.857–0.936)	0.907 (0.879–0.936)

The value was described as the predictive ability (95% CI). CI, confidence interval; AUC, area under the curve.

Supplemental Table S2. Machine learning and logistic model-based prediction models for litigation outcomes in inpatients.

	LightGBM	Decision Tree	Random Forest	Logistic Model
Accuracy	0.889 (0.853–0.926)	0.889 (0.861–0.917)	0.873 (0.845–0.901)	0.882 (0.847–0.917)
Precision	0.881 (0.850–0.913)	0.845 (0.811–0.879)	0.860 (0.832–0.887)	0.870 (0.827–0.913)
Recall	0.922 (0.868–0.977)	0.978 (0.9657–1.00)	0.918 (0.878–0.958)	0.931 (0.879–0.983)
F1 score	0.900 (0.865–0.934)	0.906 (0.883–0.929)	0.887 (0.861–0.912)	0.896 (0.866–0.926)
AUC	0.921 (0.881–0.961)	0.908 (0.875–0.941)	0.925 (0.893–0.957)	0.916 (0.881–0.851)

The value was described as the predictive ability (95% CI). CI, confidence interval; AUC, area under the curve.

Supplemental Table S3. Machine learning and logistic model-based prediction models for litigation outcomes in outpatients.

	LightGBM	Decision Tree	Random Forest	Logistic Model
Accuracy	0.706 (0.676–0.737)	0.707 (0.664–0.749)	0.736 (0.699–0.773)	0.677 (0.639–0.715)
Precision	0.730 (0.683–0.776)	0.731 (0.678–0.783)	0.739 (0.692–0.786)	0.690 (0.656–0.724)
Recall	0.729 (0.665–0.794)	0.724 (0.635–0.814)	0.795 (0.719–0.871)	0.720 (0.672–0.767)
F1 score	0.724 (0.691–0.757)	0.721 (0.672–0.770)	0.760 (0.723–0.797)	0.703 (0.669–0.737)
AUC	0.765 (0.711–0.818)	0.705 (0.649–0.760)	0.814 (0.772–0.855)	0.753 (0.701–0.806)

The value was described as the predictive ability (95% CI). CI, confidence interval; AUC, area under the curve.

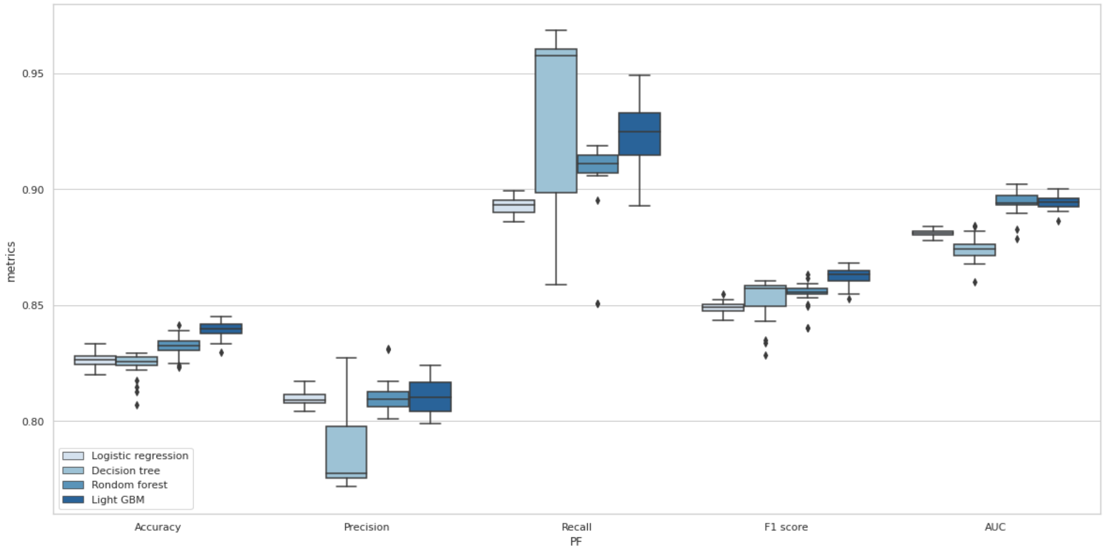

Figure S1. Performance metric evaluations of the three machine learning models and a logistic model for all cases.

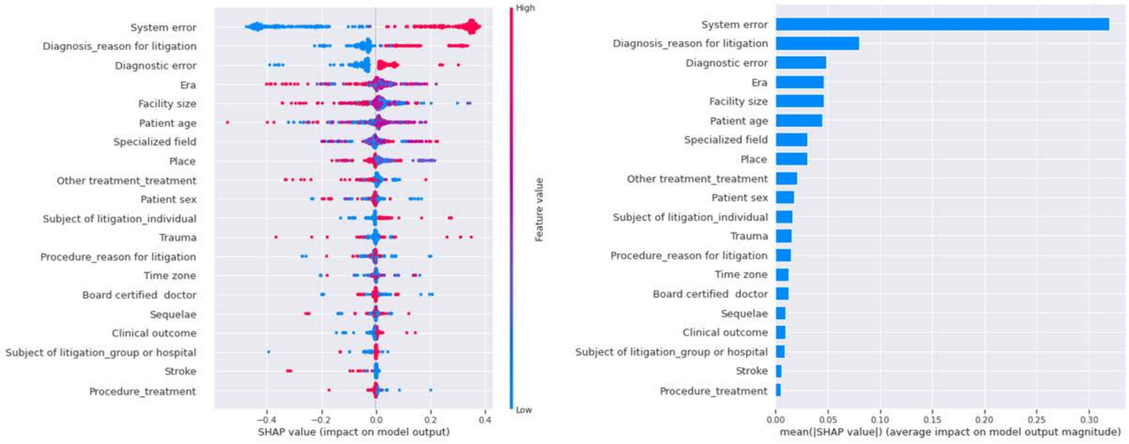

(A) (B)

Figure S2. Feature importance in decision tree with SHAP.

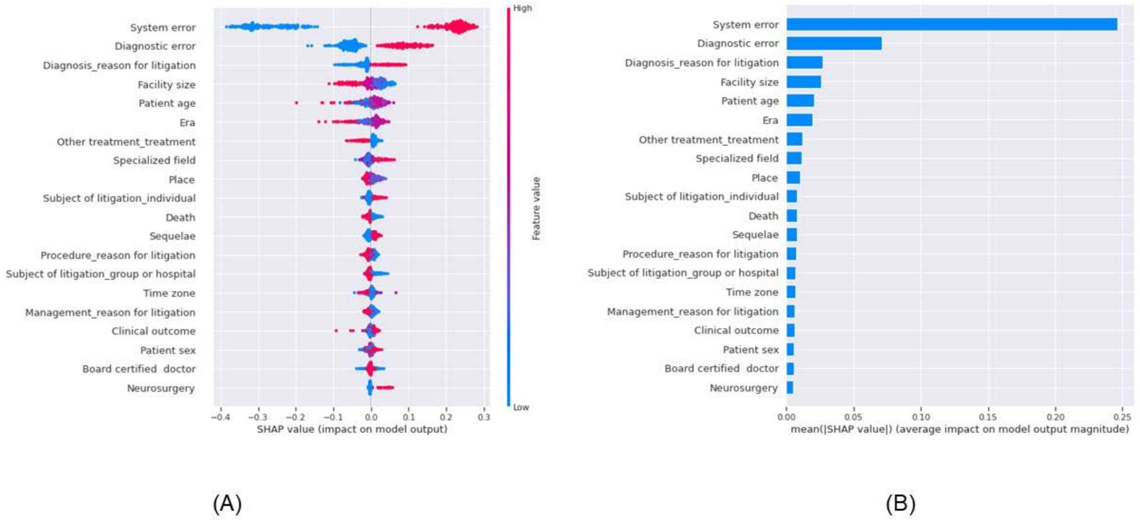

Figure S3: Feature importance in random forest with SHAP.
